# Dimensional and Volumetric Analysis of the Oropharyngeal Airway in Oral Submucous Fibrosis Patients: A Cone Beam Computed Tomography Study

**DOI:** 10.7759/cureus.115186

**Published:** 2026-08-25

**Authors:** Akash Bhokare, Amit A Mhapuskar, Anshuman Jamdade, Vaishali Jamdade, Nilofar Zaidi, Kedar Kalyanpur

**Affiliations:** 1 Oral Medicine and Radiology, Bharati Vidyapeeth (Deemed to be University) Dental College and Hospital, Pune, IND; 2 Oral Medicine and Radiology, Bharati Vidyapeeth (Deemed to Be University) Dental College and Hospital, Pune, IND; 3 Oral Medicine and Radiology, Mahatma Gandhi Dental College and Hospital, Jaipur, IND; 4 Oral and Maxillofacial Surgery, Mahatma Gandhi Dental College and Hospital, Jaipur, IND; 5 Oral Medicine and Radiology, Sinhgad Dental College and Hospital, Pune, IND

**Keywords:** airway obstruction, areca, cone beam computed tomography, oral submucous fibrosis, pharynx

## Abstract

Introduction

Oral submucous fibrosis (OSMF) is a chronic, potentially malignant disorder associated with areca nut consumption that may extend beyond the oral mucosa to affect the oropharyngeal airway, an area still inadequately characterized using three-dimensional imaging. This study aimed to comparatively evaluate oropharyngeal airway dimensions and volume across different clinical stages of OSMF and healthy controls using cone beam computed tomography (CBCT).

Methods

This prospective cross-sectional study included 80 CBCT scans: 20 healthy controls (group 1) and 60 OSMF patients equally distributed across stage I, II, and III (groups 2-4). Airway volume, minimum cross-sectional area, anteroposterior and transverse dimensions were obtained using 3D airway module across axial sections. Velar length, pharyngeal depth, and oropharyngeal width were measured on mid-sagittal CBCT sections. Data were analyzed using SPSS v26.0 (IBM Inc., Armonk, New York). Continuous variables were expressed as mean ± SD. Levene's test was used to assess homogeneity of variance across groups for each parameter. Where variances were equal (Levene's p≥0.05), one-way ANOVA followed by Tukey's post-hoc test was used for intergroup comparisons. Where variances were unequal (Levene's p<0.05), Welch's ANOVA followed by Games-Howell post-hoc test was used instead, as these methods do not assume equal variances and are more robust under heteroscedasticity. Statistical significance was set at p<0.05.

Results

All parameters showed a progressive, statistically significant reduction from controls to stage III OSMF. Airway volume decreased from 20.90±(3.06) cm³ to 5.14(±1.87)cm³ (F=162.944, p< 0.001). Minimum cross-sectional area fell from 335.93(±64.25) mm² to 62.77(±20.49) mm² (F=154.020, p<0.001). Transverse (L-R) dimension showed the steepest decline, from 28.45(±6.71) mm to 8.37(±2.54) mm (F=178.645, p<0.001), exceeding anteroposterior reduction. Velopharyngeal ratio declined from 1.07(±0.12) to 0.46(±0.15) (F=60.194, p<0.001).

Conclusions

Greater OSMF severity was associated with reduced oropharyngeal airway dimensions on CBCT, predominantly in the transverse plane. Three-dimensional CBCT assessment may provide complementary information to clinical evaluation, when imaging is appropriately justified and performed with radiation-dose optimization.

## Introduction

The oropharyngeal airway is a dynamic anatomical corridor whose patency depends on the coordinated integrity of the soft palate, pharyngeal musculature, and surrounding soft tissue [1]. It plays a central role in respiration, phonation, and deglutition, and even modest structural compromise in this region, whether from skeletal, muscular, or soft-tissue causes, can have measurable consequences for airflow and breathing efficiency [1, 2]. Because the airway is a three-dimensional structure, its accurate evaluation requires imaging capable of capturing volumetric and cross-sectional detail, rather than linear measurements alone.

Oral submucous fibrosis (OSMF) is a chronic, progressive, oral potentially malignant disorder driven predominantly by areca nut chewing, characterized by juxta-epithelial inflammation and relentless submucosal fibrosis [3, 4]. It remains endemic across South and Southeast Asia and continues to rise in prevalence with the widespread availability of commercial areca nut products [4, 5]. While OSMF has traditionally been characterized by its oral manifestations, mucosal rigidity, burning sensation, and progressive limitation of mouth opening, an expanding body of evidence indicates that the same fibrotic process extends posteriorly into the oropharynx, altering airway architecture as disease severity advances [6]. 

Early work using lateral cephalometry demonstrated soft palate and airway changes in OSMF, but this technique is inherently limited to two dimensions and cannot capture true volumetric compromise [7]. Cone beam computed tomography (CBCT) overcomes this limitation, enabling reproducible, three-dimensional volumetric and dimensional analysis at comparatively low radiation exposure [8, 9]. Using this approach, Pagare et al. demonstrated significant reductions in pharyngeal airway volume and minimum cross-sectional area in OSMF patients, worsening with clinical severity [10], while more recently, an MRI-based study by Anandan et al. confirmed measurable, stage-dependent reductions in pharyngeal airway cross-sectional area and volume even in early-stage OSMF, reinforcing the airway as a genuine, quantifiable target of disease progression rather than an incidental clinical observation [11]. Although MRI offers superior soft-tissue contrast and avoids postural artefact, it remains less accessible and more costly for routine clinical use than CBCT, which continues to be the more practical imaging modality for airway assessment in dental and maxillofacial settings [11]. 

Despite this growing evidence base, existing literature has notable gaps. Most prior studies have evaluated only a single airway parameter in isolation, typically overall airway volume or minimum cross-sectional area, without concurrently assessing anteroposterior versus transverse dimensional narrowing, oropharyngeal width, or velopharyngeal competence within the same cohort [10, 11]. Sample sizes have generally been smaller and drawn from a narrower range of disease severity, often comparing only two groups (OSMF versus controls) rather than stratifying across the full spectrum from early (stage I) to advanced (stage III) disease. Consequently, it remains unclear whether different components of airway compromise, volumetric, cross-sectional, directional (anteroposterior versus transverse), and velopharyngeal, decline in parallel as OSMF progresses, or whether they follow distinct trajectories emerging at different disease stages. We hypothesized that airway compromise in OSMF is not uniform across these parameters, and that transverse airway narrowing and velopharyngeal competence would show a distinct, later-emerging pattern of decline compared to overall airway volume and cross-sectional area. The present study was therefore designed to comparatively evaluate these dimensional and volumetric parameters of the oropharyngeal airway across four groups spanning the full clinical spectrum, healthy controls and OSMF stages I, II, and III, using CBCT, in order to test this hypothesis and provide objective, three-dimensional evidence of how airway involvement evolves with disease severity. 

## Materials and methods

The present prospective cross-sectional observational study was conducted in the Department of Oral Medicine and Radiology, Bharati Vidyapeeth (Deemed to be University) Dental College and Hospital, Pune, Maharashtra, India. After obtaining approval from the Institutional Ethics Committee. (IEC Approval No: BVDU/IEC/R4/13/23-24) The study was carried out in accordance with the ethical principles of the Declaration of Helsinki, and written informed consent was obtained from all participants before enrolment.

A total of 80 participants aged 20-45 years were included and equally allocated into four groups: group 1 (healthy controls), group 2 (stage I OSMF), group 3 (stage II OSMF), and group 4 (stage III OSMF). The calculated sample size was 20 individuals in each group, based on the following formula:

\[ n = 2(Z₁₋α/2 + Z₁₋β)²S²/Δ² \]

where Z₁₋α/2 = 1.96 (corresponding to a 95% confidence level, α = 0.05), Z₁₋β = 0.84 (corresponding to 80% power), S = 0.95 (standard deviation, derived from Singh et al.'s CBCT-based airway space study in OSMF patients), and Δ = 0.6 (expected mean difference). This yielded a calculated sample size of approximately 20 subjects per group.

Patients presenting with reduced mouth opening and a burning sensation of the oral mucosa were clinically examined, and OSMF was diagnosed and staged according to the clinical classification proposed by Nagesh and Bailoor (1993) [12]. Age- and gender-matched healthy individuals without clinical evidence of OSMF served as controls.

Inclusion and exclusion criteria

The OSMF group comprised males and females aged 20-45 years with clinically diagnosed, untreated OSMF, a habit of tobacco/gutkha chewing, and habitual swallowing of the areca nut quid juice rather than expectorating it, confirmed through habit history-taking at the time of clinical examination and applied as a strict eligibility requirement, excluding those with craniofacial deformities, malignancies or prior surgery/chemo-radiotherapy, other mucosal lesions or clefts, signs of obstructive sleep apnea, or pregnancy. The control group comprised healthy, age- and gender-matched subjects referred for CBCT for implant, orthodontic, periodontic, or minor pathology assessment, excluding those with malignancies or prior surgery/chemo-radiotherapy, degenerative diseases or clefts, craniofacial deformities, or signs of obstructive sleep apnea.

CBCT image acquisition

All participants underwent cone beam computed tomography using the Carestream CS 9600 unit (Carestream Dental, Rochester, NY, USA) with a 16 × 10 cm field of view, 90 kVp, 10 mA, and 10-second exposure time. Images were obtained with participants in an upright position, the Frankfort horizontal plane parallel to the floor, teeth in centric occlusion, lips relaxed, and breathing normally through the nose. The acquired images were reconstructed in DICOM format and analyzed using the CS 9600 Airway Analysis Tool (version 3.10.39). 

**Figure 1:**
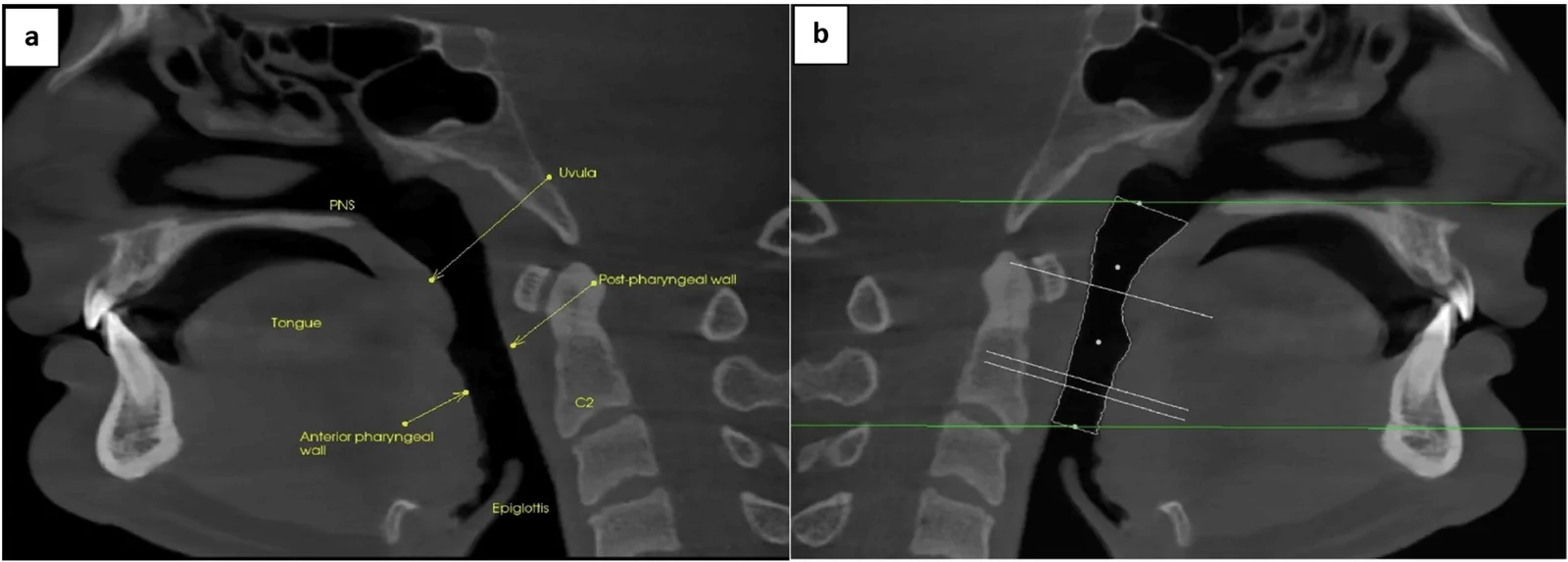
Anatomical basis for cone beam computed tomography (CBCT) airway measurements (a) Anatomical landmarks used to define the measured parameters, including the posterior nasal spine (PNS), soft palate, and posterior pharyngeal wall. (b) Superior and inferior boundaries of the airway region of interest, bounded superiorly by a line through the PNS parallel to the Frankfort horizontal plane, and inferiorly by a line through the antero-inferior point of the second cervical vertebra (C2).

**Figure 2:**
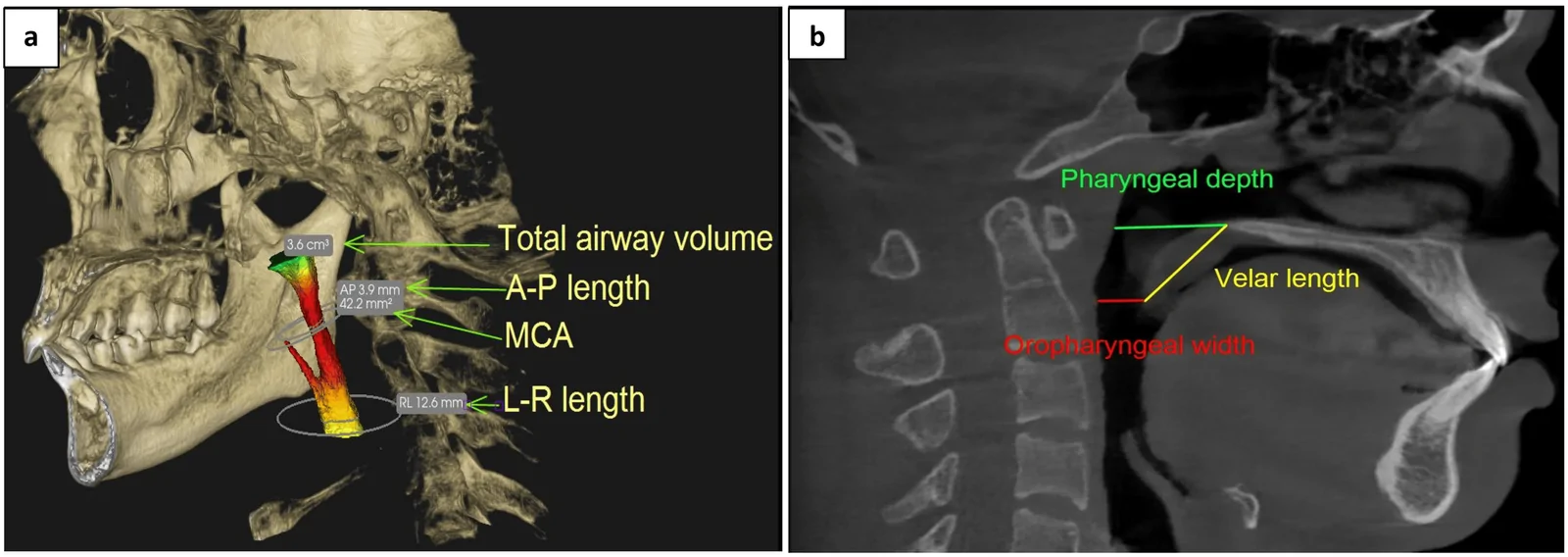
Cone beam computed tomography-based measurement protocol (a) Software interface (CS 9600 Airway Analysis Tool, version 3.10.39) showing automated computation of airway volume, minimum cross-sectional area, and minimum anteroposterior and transverse distances. (b) Linear parameters measured on the mid-sagittal section, including velar length, pharyngeal depth, and oropharyngeal airway width.

The oropharyngeal airway was segmented using the software’s airway analysis module. The region of interest was standardized using anatomical boundaries extending from the posterior nasal spine (PNS) superiorly to the anteroinferior point of the second cervical vertebra (C2) inferiorly (Figure 1). Within these limits, the software automatically calculated total airway volume (cm3), minimum cross-sectional area (mm2), minimum anteroposterior distance (A-P length in mm), and minimum left-to-right distance (L-R length in mm) as shown in Figure 1 and Figure 2. 

Oropharyngeal width was measured manually as the linear distance between the tip of the soft palate and the nearest point on the posterior pharyngeal wall (Figure 2b). To evaluate the proportional relationship between the soft palate and the pharyngeal airway, a modified velopharyngeal ratio (VL/PD) was calculated by dividing velar length by pharyngeal depth. Unlike the conventional Need’s ratio (PD/VL), which is primarily used to assess velopharyngeal insufficiency [13], the modified ratio was adopted to emphasize the relative reduction in soft palate length with respect to the available pharyngeal depth in OSMF. Pharyngeal depth (PD) was measured manually on the mid-sagittal section as the linear distance from the posterior nasal spine (PNS) to the posterior pharyngeal wall, along the palatal plane. This is distinct from the minimum anteroposterior (A-P) distance, which was derived automatically via three-dimensional segmentation as the narrowest anteroposterior width across the full axial series, and may occur at a different anatomical level than the fixed palatal-plane reference used for PD. This modification was intended to provide an anatomical index of progressive velopharyngeal morphological changes associated with fibrosis rather than a measure of functional velopharyngeal competence. Velar length and pharyngeal depth were therefore recorded solely for calculation of the modified ratio and were not analyzed as independent study variables. The anatomical landmarks, airway boundaries, and linear measurements are illustrated in Figure 1 and Figure 2.

Statistical analysis and observer reliability

Data were analyzed using SPSS v26.0 (IBM Inc., Armonk, NY, USA). Continuous variables were expressed as mean ± SD. Levene's test was used to assess homogeneity of variance across groups for each parameter. Where variances were equal (Levene's p≥0.05), one-way ANOVA followed by Tukey's post-hoc test was used for intergroup comparisons. Where variances were unequal (Levene's p<0.05), Welch's ANOVA followed by Games-Howell post-hoc test was used instead, as these methods do not assume equal variances and are more robust under heteroscedasticity. Statistical significance was set at p<0.05. A second calibrated observer independently evaluated 10% of scans to qualitatively confirm measurement consistency, and the primary observer repeated measurements after a two-week interval. Formal quantitative reliability statistics (e.g., intraclass correlation coefficient) were not calculated, as paired raw measurement values from this reliability check were not retained separately from the primary dataset

## Results

A total of 80 subjects were included in the study, equally distributed across four groups of 20 each: healthy controls (group 1), stage I OSMF (group 2), stage II OSMF (group 3), and stage III OSMF (group 4). Mean age was comparable across all four groups (one-way ANOVA, F(3,76) = 0.957, p>0.05), and sex distribution did not differ significantly between groups (χ² = 2.581, df = 3, p> 0.05), indicating that neither age nor sex was likely to confound the airway findings observed (Table 1). A male predominance was observed across all groups, most pronounced in group 4 (90%) (Table 1).

**Table 1:** Demographic characteristics of the study participants (age and gender distribution) Age comparison: one-way ANOVA, F(3,76) = 0.957, p>0.05. Sex distribution: chi-squared test, χ² = 2.581, df = 3, p>0.05.

Groups	N	Mean age (±SD) in years	Males, (n%)	Females, (n%)
Group 1 (non-OSMF controls)	20	32.50 (±5.96)	15 (75)	5 (25)
Group 2 (OSMF stage I)	20	31.30 (±6.92)	15 (75)	5 (25)
Group 3 (OSMF stage II)	20	33.55 (±8.02)	14 (70)	6 (30)
Group 4 (OSMF stage III)	20	34.85 (±6.58)	18 (90)	2 (10)

**Figure 3:**
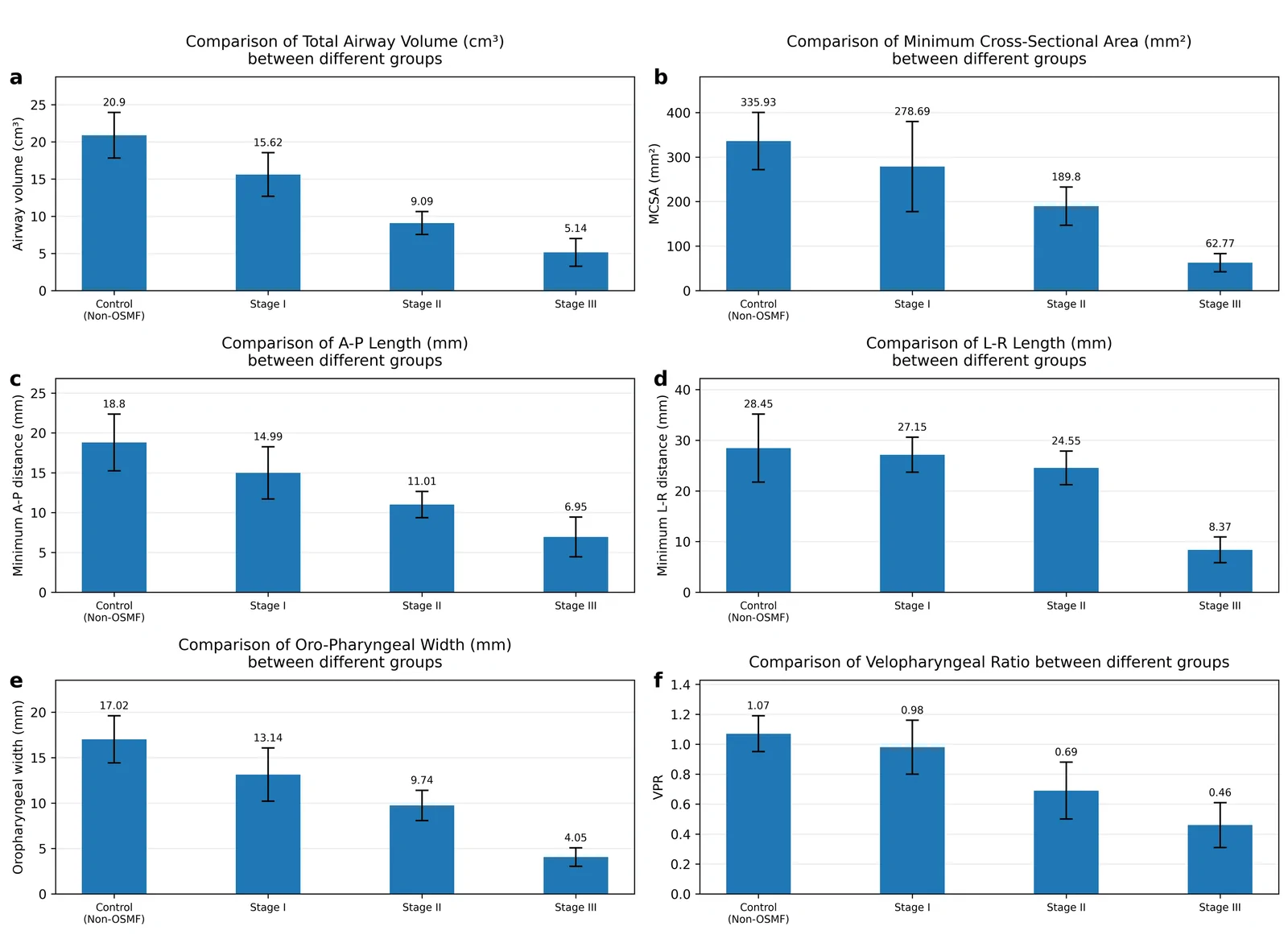
Graphical comparison of airway volume, dimensional, and velopharyngeal parameters among controls and oral submucous fibrosis patients across clinical stages I-III (a) Total airway volume; (b) minimum cross-sectional area (MCSA); (c) minimum anteroposterior (A-P) distance; (d) minimum transverse (L-R) distance; (e) oropharyngeal width; and (f) velopharyngeal ratio (VPR). Bars represent mean values, and error bars represent ±1 standard deviation (SD); values above the bars are expressed as mean ± SD.

All measured parameters showed a progressive, statistically significant decline from controls through advancing OSMF stages (Table 2, Figure 3). Airway volume decreased from 20.90(±3.06) cm³ in controls to 5.14(±1.87) cm³ in stage III (F=162.944, p<0.001). Minimum cross-sectional area fell from 335.93(±64.25) mm² to 62.77(±20.49) mm² (F=154.020, p<0.001). Minimum anteroposterior distance declined from 18.80(±3.56) mm to 6.95(±2.50) mm (F=56.335, p<0.001), while minimum transverse distance showed the steepest reduction, from 28.45(±6.71) mm to 8.37(±2.54) mm (F=178.645, p<0.001). Oropharyngeal width decreased from 17.02(±2.59) mm to 4.05(±1.02) mm (F=195.000, p<0.001). Velopharyngeal ratio declined from 1.07(±0.12) in controls to 0.46(±0.15) in stage III (F=60.194, p<0.001) (Table 2).

**Table 2:** Comparison of all the airway parameters across study groups in mean (±SD) *p<0.05. Homogeneity of variance was assessed using Levene's test; parameters with unequal variance (p<0.05) were analyzed using Welch's ANOVA with Games-Howell post-hoc test, while parameters with equal variance were analyzed using standard one-way ANOVA with Tukey's post-hoc test.

Parameter	Group 1 (control) non-OSMF	Group 2 (stage I OSMF)	Group 3 (stage II OSMF)	Group 4 (stage III OSMF)	F value	p-value	Test used
Airway volume (cm³)	20.90 (±3.06)	15.62 (±2.94)	9.09 (±1.54)	5.14 (±1.87)	162.944	<0.001*	ANOVA
Min. cross-sectional area (mm²)	335.93 (± 64.25)	278.69 (± 101.18)	189.80 (±42.80)	62.77 (±20.49)	154.020	<0.001*	Welch's
Min. A-P distance (mm)	18.80 (±3.56)	14.99 (±3.28)	11.01 (±1.65)	6.95 (±2.50)	56.335	<0.001*	Welch's
Min. L-R distance (mm)	28.45 (±6.71)	27.15 (±3.46)	24.55 (±3.32)	8.37 (±2.54)	178.645	<0.001*	Welch's
Oropharyngeal width (mm)	17.02 (±2.59)	13.14 (±2.93)	9.74 (±1.67)	4.05 (±1.02)	195.000	<0.001*	Welch's
Velar length (mm)	32.96 (±3.25)	28.08 (±6.35)	18.07 (±4.31)	10.96 (±2.88)	176.824	<0.001*	Welch's
Pharyngeal depth (mm)	30.59 (±2.61)	28.27 (±4.53)	26.23 (±3.33)	24.23 (±4.62)	9.948	<0.001*	ANOVA
Velopharyngeal ratio (VPR)	1.07 (±0.12)	0.98 (±0.18)	0.69 (±0.19)	0.46 (±0.15)	60.194	<0.001*	ANOVA

Post-hoc comparisons (Tukey's for airway volume, pharyngeal depth, and VPR; Games-Howell for all other parameters, following Levene's test for variance homogeneity) showed that most intergroup differences reached statistical significance. Exceptions included minimum cross-sectional area between group 1 and group 2 (p>0.05), and minimum transverse (L-R) distance between group 1 and group 3 (p>0.05) and between group 2 and group 3 (p>0.05), indicating that transverse and cross-sectional narrowing become statistically distinguishable from controls only from stage II or III onward. Minimum A-P distance, oropharyngeal width, and velar length showed significant differences across all pairwise comparisons (p≤0.023).

## Discussion

The demographic profile of this study showed a comparable mean age across all four groups (ranging from 31.30 to 34.85 years), a narrow spread that is unlikely to be clinically meaningful given that age-related changes in pharyngeal airway dimensions and soft tissue laxity typically become relevant over much wider age gaps or in older populations. This close demographic matching strengthens confidence that the airway differences observed between groups reflect OSMF-related fibrotic change rather than age-associated anatomical variation. A male predominance was seen across all groups, most pronounced in stage III (90%), consistent with the well-established gender skew in areca nut chewing habits and OSMF prevalence generally. This is a relevant methodological point given that Pagare and Kale explicitly identified broad age range and uneven gender distribution across their OSMF and control groups as a limitation affecting their ability to assess demographic effects on airway parameters. In contrast, the tighter demographic comparability achieved in the present study likely improved the sensitivity of the intergroup comparisons reported here [14].

Airway volume and minimum cross-sectional area both declined progressively and significantly from controls through stage III OSMF. This is well supported by the broader OSMF-airway literature: Agrawal et al. were among the first to report changes in upper, middle, and lower airway dimensions in OSMF using digital cephalometry, attributing this to soft tissue modification from fibrosis [15]. Singh et al., using CBCT volumetric assessment, similarly reported reduced airway volume in OSMF patients [16], as did Pagare et al.'s CBCT study [10] and Panwar et al., who reported a significant reduction in airway volume in OSMF patients (17.16 cm³) compared to healthy controls (22.04 cm³), attributing this to progressive fibrosis of the pharyngeal walls and soft palate musculature [17]. Anandan et al., using MRI, confirmed significant reductions in pharyngeal cross-sectional area and volume, particularly in stage III disease [11]. Minimum cross-sectional area is arguably the most functionally consequential parameter, since airway resistance is governed principally by the narrowest point along the airway; its marked reduction here, from 335.93 mm² in controls to 62.77 mm² in stage III, is consistent with this broader body of evidence pointing toward genuine, clinically meaningful airway compromise in advancing disease.

Both anteroposterior and transverse dimensions declined significantly with advancing OSMF stage, with transverse narrowing notably more severe by stage III than anteroposterior narrowing. Byatnal et al., in a preliminary cephalometric evaluation of pharyngeal airway in OSMF, similarly reported reductions in airway dimensions with disease progression [18], and Agrawal et al.'s cephalometric analysis reported comparable dimensional narrowing across upper, middle, and lower airway measurements [15]. However, the observed differences in transverse airway dimensions, which were more apparent at stage III, may suggest a transverse component to airway narrowing that has not been explicitly characterized in much of the existing literature. Previous studies have generally assessed airway narrowing using combined or overall measurements rather than separately examining the anteroposterior and transverse dimensions. This observation should therefore be considered hypothesis-generating, particularly because the minimum anteroposterior distance, minimum lateral-to-right distance, and minimum cross-sectional area may occur at different axial levels within the airway.

Velopharyngeal ratio, a composite indicator of velopharyngeal competence, declined significantly from 1.07 in controls to 0.46 in stage III, indicating progressive shortening of the soft palate relative to pharyngeal depth as disease advances. This is consistent with Shah and Shah's radiographic evaluation of soft palate morphology in OSMF [7], Nerkar et al.'s comparative cephalometric analysis showing soft palate shortening between OSMF and normal individuals [19], and Gupta et al.'s CBCT-based assessment of soft palate morphology, which similarly reported altered soft palate dimensions with disease [20]. Together with the transverse airway findings above, this suggests that velopharyngeal structures, not just the bony/airway boundary itself, undergo measurable geometric change as OSMF progresses, likely reflecting fibrotic contracture reshaping the soft palate rather than uniform shrinkage alone.

A notable point of contrast with the literature is that every parameter measured in the present study reached statistical significance, whereas Pagare and Kale's recent CBCT study found no significant difference in total airway volume between OSMF and controls, or across stages, despite finding significant changes in soft palate length, width, and superior airway space [14]. Several methodological differences plausibly explain this divergence. First, airway volume in the present study was computed using the CS 9600 Airway Analysis Tool's automated segmentation, whereas Pagare and Kale used manual, slice-by-slice painting in ITK-SNAP, a method the authors themselves acknowledged introduces operator-dependent variability [14], consistent with Schendel and Hatcher's observation that automated three-dimensional airway analysis improves reproducibility over manual segmentation approaches [21]. Second, the present study included stage I OSMF alongside stages II and III, capturing the full spectrum of disease and enabling detection of a clear, early-emerging linear trend, whereas Pagare and Kale's cohort began at stage II, potentially missing the earliest phase of airway change and analyzing a narrower disease range with unevenly distributed subgroup sizes. Third, differing staging classifications across studies - Nagesh and Bailoor's system [12] used here versus Kiran Kumar et al.'s clinico-histopathological staging used elsewhere in this literature [22] - may themselves group patients differently by underlying fibrotic severity, further contributing to inconsistent findings across institutions. Fourth, the present study specifically selected patients who habitually swallowed the areca nut quid juice rather than expectorating it, a habit pattern not specified in most prior OSMF-airway studies. Given this predefined habit characteristic, sustained mucosal and oropharyngeal exposure to arecoline-laden saliva in quid-swallowers may represent a possible mechanism contributing to more extensive posterior fibrotic involvement; however, this mechanism was not directly evaluated in the present study and should therefore be regarded as a tentative hypothesis rather than an established explanation for the observed airway findings. Rather than undermining the present findings, this divergence highlights a genuinely unresolved methodological question in the field: whether airway volume changes in OSMF are consistently detectable across different segmentation approaches, staging systems, and habit characteristics, or whether they may be influenced by these methodological factors.

Taken together, these findings indicate that OSMF is associated with progressive, multi-parameter compromise of the oropharyngeal airway, encompassing volume, cross-sectional area, dimensional narrowing, and velopharyngeal competence, with transverse constriction emerging as a particularly severe late-stage feature. While this pattern is well supported by a growing, if still occasionally inconsistent, body of CBCT and MRI-based literature, the discrepancies noted above underscore the need for standardized segmentation protocols and staging criteria before airway assessment can be reliably incorporated into routine OSMF evaluation across institutions. 

Limitations

This study's cross-sectional design captures differences between groups at a single point in time rather than confirming a true within-patient trajectory of airway change as OSMF progresses. The sample was drawn from a single institution, which may limit generalizability, and the unequal sex distribution across the study groups may have introduced potential sex-related confounding. In addition, clinical staging was used without histopathological confirmation of the extent or severity of fibrosis. Although homogeneity of variance was assessed as part of the statistical analysis, the reliability of manual landmark identification may still be affected by observer-dependent variability; reliability statistics have therefore been reported to assess measurement consistency. Although measurement consistency was assessed through repeat measurements and independent evaluation, formal quantitative reliability statistics such as ICC were not calculated because paired raw measurements were not retained. Future studies should incorporate ICC with 95% confidence intervals for continuous CBCT measurements. Structural airway narrowing was evaluated independently of functional respiratory outcomes such as polysomnography, and the present study did not assess whether the observed anatomical differences translate into increased airway resistance or respiratory dysfunction. Furthermore, CBCT provides a static assessment of the airway at a single respiratory phase and therefore cannot capture the dynamic changes in airway dimensions that occur throughout the respiratory cycle. Future multicentric, longitudinal studies incorporating histopathological correlation, functional respiratory assessment, and dynamic or respiratory-phase-specific airway evaluation are warranted to further clarify the clinical significance of these findings.

Future prospects

Future longitudinal studies following the same patients across disease stages are needed to determine whether the observed airway differences represent longitudinal changes in airway dimensions rather than cross-sectional differences between groups. Correlation of structural airway measurements with functional respiratory outcomes, together with larger multicentric samples, standardized segmentation and measurement protocols, and quantitative reliability assessment, may help clarify the inconsistencies in the existing OSMF-airway literature. Further studies incorporating dynamic or respiratory-phase-specific airway assessment may also provide greater insight into the functional significance of these anatomical findings.

## Conclusions

This study demonstrated that more advanced stages of oral submucous fibrosis were associated with reduced oropharyngeal airway dimensions, including airway volume, minimum cross-sectional area, anteroposterior and transverse dimensions, oropharyngeal width, and velopharyngeal competence as reflected by the corresponding ratio. Transverse constriction emerged as a particularly severe and late-appearing feature of advanced disease, distinguishing it from the earlier, more gradual decline observed in volumetric and cross-sectional parameters. Cone beam computed tomography enabled three-dimensional assessment of these airway changes, supporting its potential utility as an adjunct to conventional clinical evaluation. Its use should, however, be guided by appropriate clinical justification and optimization of radiation exposure in accordance with the As Low As Reasonably Achievable (ALARA) principle. These findings support the growing recognition that OSMF is not a disease confined to the oral mucosa, but one with measurable, progressive structural consequences for the oropharyngeal airway, consequences that may benefit from earlier identification and closer clinical attention as disease severity advances.
